# Case report of a 65-year-old man with biatrial metastatic localisation from poorly differentiated cutaneous squamous cell carcinoma

**DOI:** 10.3332/ecancer.2019.977

**Published:** 2019-11-19

**Authors:** Lorenzo Dottorini, Italo Sarno, Pasquale Scopelliti, Gianluca Cotroneo, Maribel Duluc, Alessandro Iaculli, Nicola Giuntini, Federica Brena, Giuseppe Nastasi

**Affiliations:** 1 Oncology Unit, Medical Sciences Department, ASST Bergamo Est, Alzano Lombardo, BG 24022, Italy; 2 Cardiology Unit, Medical Sciences Department, ASST Bergamo Est, Alzano Lombardo, BG 24022, Italy

**Keywords:** cutaneous, skin, carcinoma, heart, atrial metastases, cancer, biatrial

## Abstract

We report the case of an immunocompetent 65-year-old man affected by cutaneous squamous cell carcinoma (cSCC) with lung and biatrial metastatic localisation. In May 2018, the patient underwent lower limb amputation due to the finding of a large ulceration which upon biopsy was found to be a poorly differentiated squamous cell carcinoma (SCC), ulcerated, full-thickness infiltrating from the skin to the underlying bone tissue. After 1 month, a radiological restaging found multiple pulmonary localisations and a right-atrial metastatic localisation. The patient was then studied in-depth and a transesophageal echocardiogram found that the patient had two 2 and 5 cm metastatic localisations in the left atrium and a 3-cm metastatic localisation in the right atrium. Informed about the clinical situation and about the risks of a chemotherapeutic treatment, the patient decided not to start any treatment. This case represents, to our knowledge, the only case of a biatrial metastatic localisation from cSCC and is representative of how cardiac symptoms and signs in patients affected by this disease must be evaluated.

## Background and introduction

Cutaneous squamous cell carcinoma (cSCC) is the second most common form of nonmelanoma skin cancer after basal cell carcinoma. [1] Its origin is the malignant proliferation of epidermal keratinocytes due to multiple biological events [2, 3]. Previously reported findings showed how immunosuppressed patients tend to develop multiple and more aggressive skin cancer. Patients undergoing solid organ transplantation have a 65-fold higher risk of developing cSCC than the general population [4–6]. This tumour has a high rate of recurrence and distant metastasis, especially for those patients with tumours involving the lips and ears with frequent involvement of lateral-cervical, submandibular, submental and intraparotid lymph nodes [7]. Cardiac metastatic localisation from cSCC is an extremely rare event and very poor scientific evidence is available. We found no evidence of a double atrial localisation. The case report we described could be the first scientific evidence of this rare occurrence and could help clinicians to evaluate all cardiac symptoms and signs in cSCC patients.

## Case presentation

**Figure 1:**
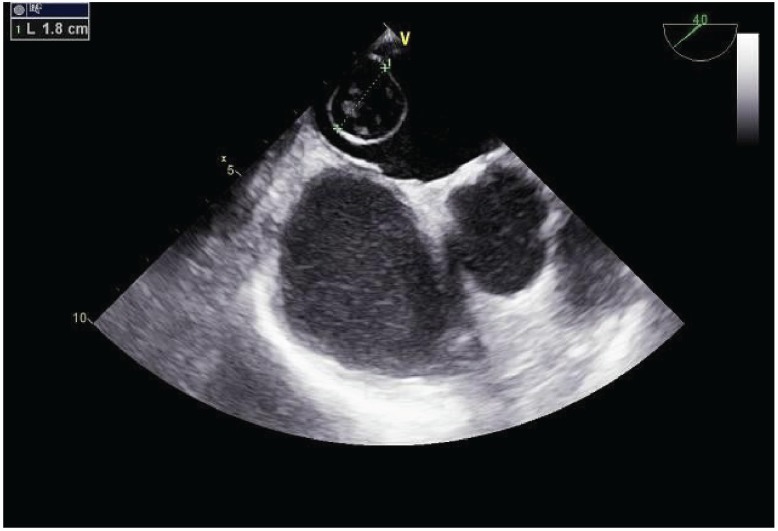
Transesophageal echocardiogram of the mass in the left atrium.

**Figure 2:**
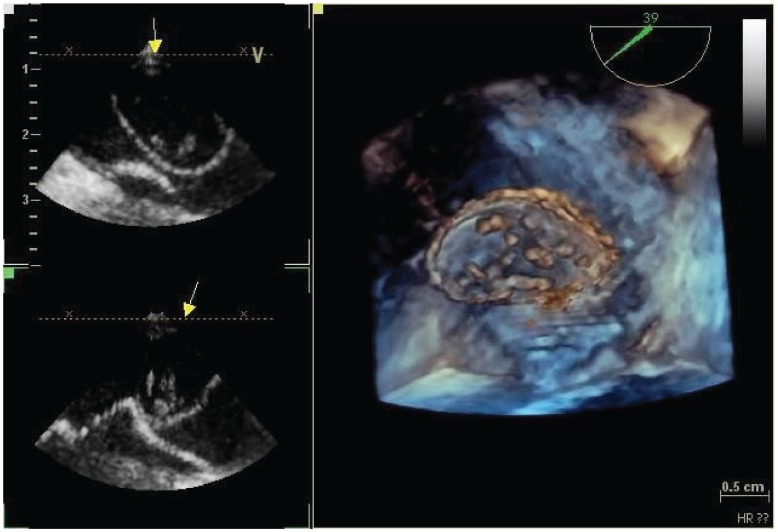
Tridimensional transesophageal echocardiogram of the mass in the left atrium.

**Figure 3:**
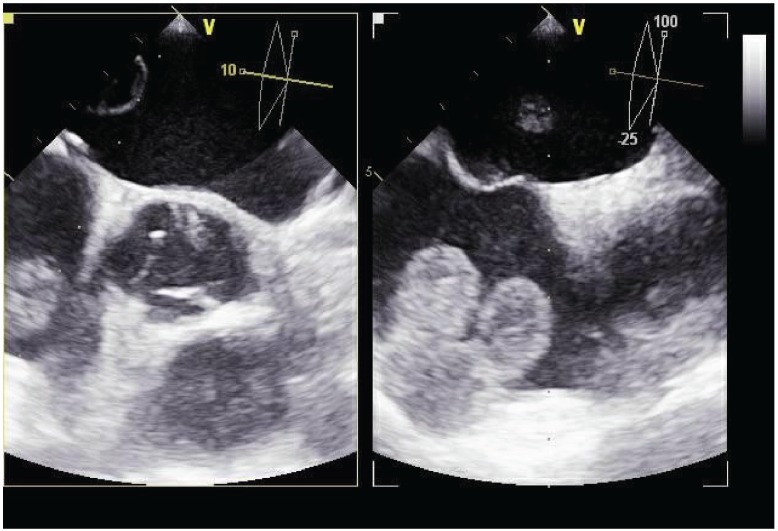
Transesophageal echocardiogram of the mass in the right atrium.

**Figure 4:**
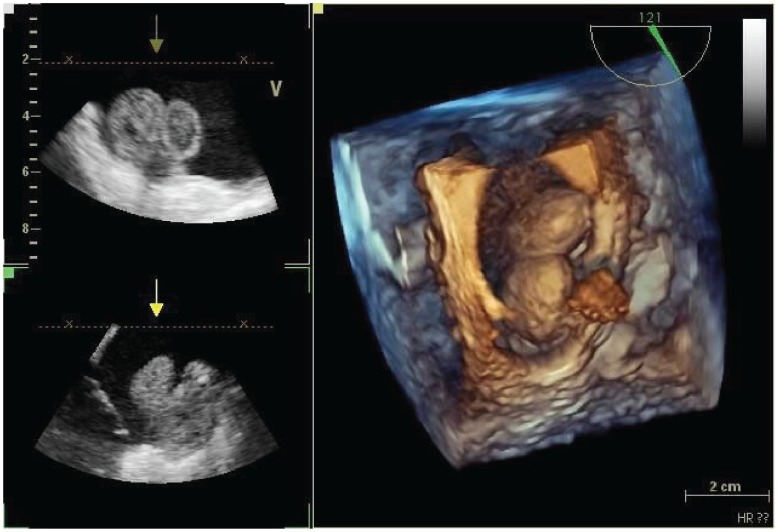
Tridimensional transesophageal echocardiogram of the mass in the right atrium.

**Figure 5:**
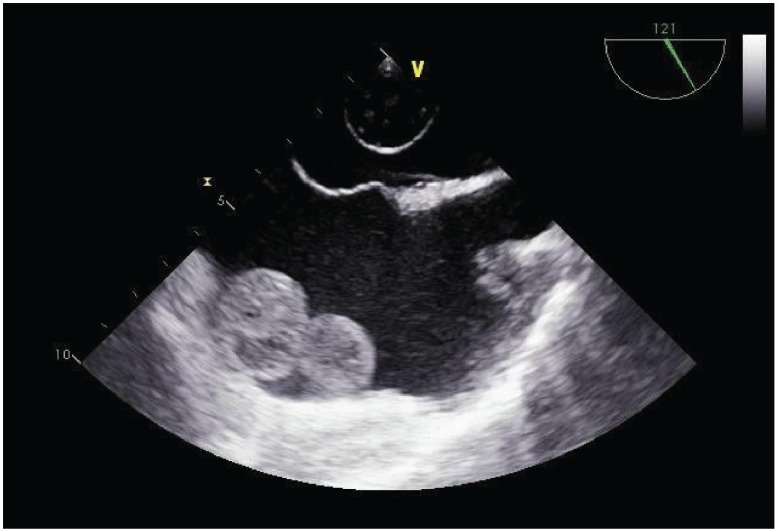
Transesophageal echocardiogram of the mass both in the right and left atria.

In May 2018, an immunocompetent 65-year-old man with no familial history of skin cancer was recovered due to a large ulceration involving the proximal portion of the right leg causing hypofunctionality of the limb and osteomyelitis with loss of neuronal substance. A cutaneous biopsy demonstrated an SCC. The ulceration was too large to be subjected to local excision; therefore, after a complete radiological staging with chest and abdomen computed tomography that did not show distant metastases, amputation of the middle third of the right leg was made. The definitive histological examination showed an ulcerated area of 13 cm, necrotic, haemorrhagic, incorporating and full-thickness infiltrating from skin to the underlying bone tissue, compatible with poorly differentiated SCC. For persistence of secretions, the wound was revised and cleaned several times. One month after amputation, a radiological restaging with chest and abdomen computed tomography showed multiple bilateral lung lesions compatible with distant metastases and a dubious intracardiac nodularity. The patient was then studied for a possible infectious disease but all the exams (including tuberculosis screening) were negative. We performed a fibrobronchoscopy with biopsy of a mediastinal lymph node and bronchioloalveolar lavage, resulting in positive SCC in both samples. The following positron emission tomography highlighted multiple lung localisations (Standardised uptake value 20), widespread skeletal accumulation referable to osteomidullary activity compatible with substitutive meaning and a left and right atrial nodularity compatible with metastases (standardised uptake value 7, 4). Echocardiography revealed a solid lesion on the atrial side of the anterior tricuspid flap; the ejection fraction determined to be 85%. A transesophageal echocardiography documented in the right atrium a voluminous tripartite formation with a sessile spherical base of 3 cm in diameter and two other non-homogeneous mobile formations of 2 and 5 cm in diameter, each comes into contact with the atrial margin of the tricuspid valve. In the left atrium, evidence of a roundish 2 cm diameter formation anchored by a peduncle to the atrial roof was compatible with metastases (Figures 1–5). A cardiac surgery consultation determined that the extent of the disease, including at the pulmonary level, suggested that surgical intervention was inappropriate. A cardiac nuclear magnetic resonance was unsuccessful since the patient experienced rapid dyspnoea that arose from lying in a supine position. Despite the extent of the disease and the cardiac involvement, considering the young age of the patient and the conserved cardiac function, we proposed a platinum-based chemotherapeutic treatment; however, when informed of the possible risks and benefits of the treatment, the patient preferred not to undertake any oncological treatment.

## Discussion

Cutaneous carcinomas (basal and squamocellular) derive from epidermal keratinocytes and skin appendages and represent over 90% of malignant skin tumours [8]. Exposure to UV radiation (especially UVB) is the main risk factor for these neoplasms [9] and the initiator of genetic damage at the base of their development, both through the production of reactive oxygen compounds (ROS), and through the loss of heterozygosity (LOH) of tumour suppressor genes [10]. Other factors involved in the genesis of these lesions are consumption of tobacco (for the squamocellular forms), arsenic, vinyl chloride, polycyclic aromatic hydrocarbons, alkylated agents and exposure to vapours of petrol [11]. Lower risk factors such as immunosuppression (favoured by UVB) and HPV infection (which is often found in immunosuppressed patients) appear in connection with this type of carcinoma. Tumour diameter (>2.0 cm) and perineural involvement (> 0.1 mm) are mostly associated with disease-specific death and nodal metastases [12]. Cardiac metastases from cSCC are an event extremely rare and few data are available in the scientific literature. Mackenzie *et al* [13] published a case report about a myocardial metastasis of cSCC in a renal transplant recipient whose cause of death was pulmonary thromboembolism. Boukhalil *et al* [14] reported a case of cSCC with pericardial metastases, whereas Hunter *et al* [15] reported a case of right ventricular inflow tract obstruction secondary to cSCC.

## Conclusion

To the best of our knowledge, there are no cases in the scientific literature reporting a metastatic localisation of cSCC in both cardiac atria. Our case report underlines how patients with advanced cancer, including cSCC, presenting cardiac signs and symptoms such as fatigue, arrhythmia, congestive heart failure or cardiac tamponade must always be suspected of having tumour involvement of the heart.

## Conflicts of interest

The authors declare that they have no conflicts of interest.

## Funding statement

No funding was received.
